# Neuroprotective Properties of the Marine Carotenoid Astaxanthin and Omega-3 Fatty Acids, and Perspectives for the Natural Combination of Both in Krill Oil

**DOI:** 10.3390/nu6031293

**Published:** 2014-03-24

**Authors:** Marcelo P. Barros, Sandra C. Poppe, Eduardo F. Bondan

**Affiliations:** 1 Institute of Physical Activity and Sports Science (ICAFE), Cruzeiro do Sul University, 01506-000 São Paulo, Brazil; E-Mail: sandra.poppe@cruzeirodosul.edu.br; 2 Graduation Program in Health Sciences, Cruzeiro do Sul University, 01506-000 São Paulo, Brazil; E-Mail: eduardo.bondan@cruzeirodosul.edu.br; 3 Department of Veterinary Medicine, University Paulista (UNIP), 01506-000 São Paulo, Brazil

**Keywords:** seafood, neurodegenerative disorders, antioxidant, anti-inflammatory, cognition, oxidative stress, natural products, Alzheimer’s, Parkinson’s, hormesis

## Abstract

The consumption of marine fishes and general seafood has long been recommended by several medical authorities as a long-term nutritional intervention to preserve mental health, hinder neurodegenerative processes, and sustain cognitive capacities in humans. Most of the neurological benefits provided by frequent seafood consumption comes from adequate uptake of omega-3 and omega-6 polyunsaturated fatty acids, *n*-3/*n*-6 PUFAs, and antioxidants. Optimal *n*-3/*n*-6 PUFAs ratios allow efficient inflammatory responses that prevent the initiation and progression of many neurological disorders. Moreover, interesting *in vivo* and clinical studies with the marine antioxidant carotenoid astaxanthin (present in salmon, shrimp, and lobster) have shown promising results against free radical-promoted neurodegenerative processes and cognition loss. This review presents the state-of-the-art applications of *n*-3/*n*-6 PUFAs and astaxanthin as nutraceuticals against neurodegenerative diseases associated with exacerbated oxidative stress in CNS. The fundamental “neurohormesis” principle is discussed throughout this paper. Finally, new perspectives for the application of a natural combination of the aforementioned anti-inflammatory and antioxidant agents (found in krill oil) are also presented herewith.

## 1. Oxidative Stress in Brain Tissues

Oxidative stress is defined as a condition in which oxidant generation overwhelms antioxidant defenses and is largely implicated in the pathogenesis of many neurologic and psychiatric diseases [1,2,3]. Neurons and, secondarily, glial cells, have high demand for O_2_ to produce, via mitochondria, the energy (ATP) required to maintain ionic homeostasis, synthesis of key molecules and housekeeping cell activities of the central nervous system (CNS) [4]. Under physiological conditions, 1%–2% of the O_2_ consumed by mitochondria is normally converted into reactive oxygen species (ROS), like the superoxide anion (O_2_^•−^) and hydrogen peroxide (H_2_O_2_) [4]. Although mitochondria are the main intracellular sources of ROS, other compartments also contribute to impose oxidative stress in cells, particularly the cytosol (e.g., xanthine oxidase activity) and the plasma membrane enzyme NADPH oxidase (NOX) [3]. There are seven NOX genes already identified, although only NOX1, NOX2, and NOX4 were effectively found in neurons, astrocytes, and microglia [5].

Nitric oxide (NO•) is considered the precursor of the more aggressive reactive nitrogen species (RNS) in intracellular compartments or extracellular fluids. Highly diffusible throughout membranes, NO• is a key biological messenger in the CNS and is produced via three different enzymatic paths, including neuronal NO• synthase (nNOS, type I), inducible NO• synthase (iNOS, type II, present in microglial cells and macrophages) and endothelial NO• synthase (eNOS, type III). Whenever NO• production increases after xNOS activation, it promptly reacts with the ubiquitous O_2_^•−^ radical to form peroxynitrite (ONOO^−^), in one of the fastest reactions to occur in biological systems (k_1_ ~3–5 ° 10^9^ M^−1^ ·s^−1^) [4]. Peroxynitrite is not a free radical *per se* but can oxidize lipids, DNA, and proteins directly or via derivative radicals, such as nitrogen dioxide (NO_2_^•^) or the carbonate radical (CO_3_^•−^) [6].

Enhanced ROS/RNS generation, in addition to inefficient antioxidant defenses, could be detrimental to cell survival, especially in neurons, because mitochondrial function depends significantly on an appropriate redox balance [7]. An interesting and relatively novel concept stipulates that thiol-dependent redox switches could sense, or even determine, the fates of different cells, including those of the animal and human CNS. Therefore, three processes could be triggered in the resting G0 stage by unbalanced redox conditions: (i) duplication/differentiation, when more reduced conditions are present due to enhanced NADPH biosynthesis; (ii) programmed cell death (apoptosis), induced by extrinsic or intrinsic moderately high oxidative stress; or (iii) uncontrolled cell death (necrosis), when oxidative conditions are extreme [8]. It is worthwhile to note that physiological and metabolic adaptations through antioxidant responses are expected to occur under milder oxidative challenge, which does not necessarily lead to abrupt changes in cell function or fate. Strong evidence proves that moderate but regular exercise, for example, provides health benefits in humans by inducing such positive antioxidant responses [9]. Although neuronal death may occur by necrosis or apoptosis, the second form unquestionably represents the predominant type in chronic neurodegenerative diseases. Thus, oxidative stress and apoptosis are apparently linked in the pathophysiology of many neurodegenerative diseases [4,7].

The human brain is abnormally prone to oxidative stress for several reasons: (i) intense mitochondrial activity in neurons generates massive amounts of ROS/RNS; (ii) high concentrations of radical-sensitive polyunsaturated fatty acids are present in neuronal membranes; (iii) redox-active iron ions accumulate in the mammalian brain; (iv) neurotransmitters are prone to auto-oxidation, resulting in the production of neurotoxic metabolites (e.g., 6-hydroxydopamine from dopamine); and (v) the H_2_O_2_-removing enzyme catalase (CAT) exhibits lower activity in most brain regions [10,11].

Besides the eventual neuronal damage induced by ROS/RNS, insults in the CNS invariably result in the activation of glial cells (particularly astrocytes and microglia) at the sites of injury. Immune mediators (e.g., NO•, ROS/RNS, pro-inflammatory cytokines, and chemokines) released by activated glial cells are currently considered putative neurotoxins [1]. Pro-inflammatory cytokines such as tumor necrosis factor-α (TNF-α) and interleukin-1β (IL-1β) are upregulated within hours in ischemic brain lesions [12]. Although glial responses and oxidative insults significantly differ between brain regions and could thereby result in selective neuronal death and distinct resistance to ischemic damage in neighboring areas, hallmarks of inflammation and redox metabolism are necessary for a precise diagnosis of the initiation and progress of neurodegenerative diseases [13,14,15].

## 2. Antioxidant Defenses and “Neurohormesis”

Antioxidants are essential for life and optimal health. The link between sufficient antioxidant intake and healthy development from child to adulthood, high-quality aging, preserved cognition, and longevity is increasingly supported by scientific findings and longitudinal clinical evaluations. Following formal definitions, an antioxidant is “any substance that, when present at low concentrations compared to those of a putative oxidizable target, significantly delays or prevents oxidation” [16]. Antioxidant defenses are composed of enzymatic and nonenzymatic compounds that decrease the steady-state concentrations of ROS/RNS responsible for oxidative damage to vital biomolecules. Cellular antioxidants include low-weight molecules, such as glutathione (GSH), carotenoids (such as β-carotene and lycopene), ascorbic acid, urate, and tocopherols, but also macromolecules (e.g., bilirubin), proteins (e.g., ferritin and transferrin), and enzymes that remove ROS/RNS, such as superoxide dismutase (SOD), glutathione peroxidase (GPX), and catalase (CAT) [17].

Based solely on *in vitro* properties, most antioxidants in the plasma effectively prevent and/or scavenge ROS/RNS when present in the 50–300 μM range (e.g., tocopherols, ascorbate, and urate). However, in many cases, much lower concentrations of antioxidants (or phytochemicals) also exhibited positive neuroprotective activity [18]. Flavonoids and some carotenoids (excepting the prevalent β-carotene) are usually found in the plasma of treated animals at low concentrations (1–5 μM), although significant improvement of cognitive functions was also observed in the clinical context [19,20,21,22].

Scientific evidence showed that many dietary phytochemicals that promote the health of the CNS might act, in fact, as moderate prooxidant agents [18]. By imposing mild stress on neural cells, these phytochemicals induce positive antioxidant responses—especially increments in GSH and other thiol pools—that increment the scavenging capacity of CNS. Therefore, the previously acquired thiol increment enhances the ability of neural tissues to cope with more severe oxidative stress provided by infectious agents or pathological processes, increasing resistance against neurodegenerative diseases. With respect to the CNS, this mechanism is known as the “neurohormesis” principle, and is usually associated with enhanced neurogenesis, synaptic plasticity, and resistance to oxidative injury imposed by neurodegenerative diseases [18].

**Figure 1:**
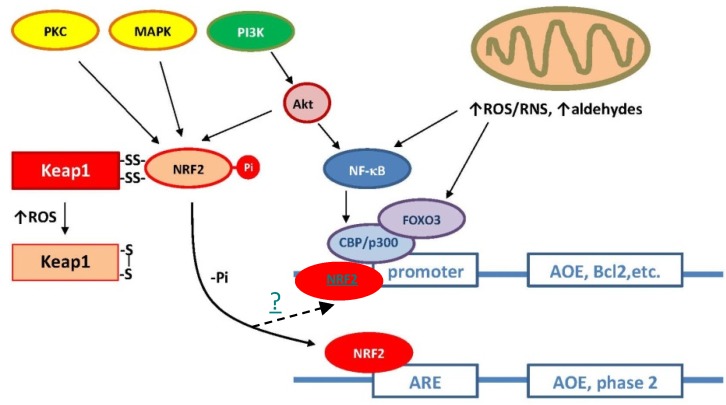
Main antioxidant signaling pathways in mammals. Under normal conditions (elevated intracellular reduced potential), nuclear factor erythroid 2-related factor 2 (Nrf2) is stabilized through binding to Keap-1 in the cytoplasm. Under oxidative stress, thiol groups in Keap-1 are oxidized (e.g., S-S crosslinks) causing the dissociation of Nrf2, translocation to the nucleus, and binding to the antioxidant-responsive elements (ARE). Depending upon the binding site present in the promoter region, different antioxidant genes are induced.

Transcription factors that mediate neurohormesis include NF-κB, Kelch-like ECH-associated protein 1 (Keap1), cAMP-response-element-binding protein (CREB), nuclear factor erythroid 2-related factor 2 (Nrf2), and hypoxia-inducible factor 1 (HIF1) [23,24,25,26]. Many of these transcription factors act on different antioxidant-responsive elements (AREs), depending on the activated pathway. The Keap1-Nrf2-ARE signaling pathway can be considered the main axis of the adaptive response against oxidant stress [27]. At the molecular level, the binding of Nrf2 to AREs induces the transcription of antioxidant genes including SOD, GPX, CAT, glutathione-S-transferase (GST), and GSH biosynthetic enzymes. Several other cytoprotective genes (for the expression of hemeoxygenases, NQO1, HO-1, *etc.*) are also activated by the Keap1-Nrf2-ARE pathway (Figure 1). Accordingly, neuroprotection and the Keap1-Nrf2-ARE signaling axis have been found to be strongly correlated in *in vitro*, *in vivo*, and clinical studies [28].

Therefore, a better comprehension of “pharmacological-therapeutic windows” becomes necessary to fully appreciate the “neurohormesis” principle and, thereby develop maximally efficient antioxidant therapies against ROS/RNS-mediated neurodegenerative processes; these windows include the following: (i) quality and quantity of antioxidants in foods compared to a specific supplementation; (ii) bioavailability of antioxidants in the human digestive tract; (iii) absorption rates; (iv) hepatic biotransformation and metabolism; (v) pharmacodynamics and target-tissue accumulation; and (vi) excretion rates [29,30].

## 3. Redox Imbalances and Neurodegenerative Pathologies

Almost all CNS disorders, including multiple sclerosis (MS), Alzheimer’s disease (AD), and Parkinson’s disease (PD), are associated with some degree of neuroinflammation and related redox imbalances. In this context, microglial cells have been proposed as important contributors in chronic neurodegeneration and oxidative stress as they produce and release a variety of cytoactive factors, including ROS and NO•, glutamate, and cytokines [31]. In AD, for example, microglial cells surrounding the senile plaques express major histocompatibility complex (MHC) class II molecules, a feature of antigen-presenting cells. Aβ peptides or fragments of amyloid precursor protein increase O_2_^•−^ production in rat peritoneal macrophages and cultured rat microglia [32]. NO• formation is also induced in mouse microglia by Aβ peptides [33]. As neurons are post-mitotic cells, their general incapability to divide explains some aging and degeneration-related dysfunction, considering that the capacity for neuronal replacement is very limited (although many parts of the CNS have considerable neuronal redundancy) [4]. Furthermore, considering the “neurohormesis” principle, neuronal cells are apparently functional under restricted redox conditions, in contrast to more versatile cells, such as hepatocytes and myocytes. In this respect, hypoxia and ischemia-reperfusion processes could be literally lethal for many neuronal cells in the affected brain region [34].

Astrocytes can survive chronic hypoxia as well as long periods of ischemia, *i.e.*, hypoglycemia and anoxia. On the other hand, oligodendrocytes are preferentially injured over astroglia during chronic hypoxia, reperfusion following ischemia, hypoglycemia, or uncoupling of oxidative phosphorylation, because they are more susceptible to oxidative stress due to their lower GSH and higher iron content [35]. It is well known that iron ions play a pivotal role in the generation of highly reactive radicals, such as HO•, ROO•, and RO• [8]. Oligodendrocyte precursor cells (OPCs) and astrocytes in 2-week-old rat primary glial cultures survived 24 h of anoxia, suggesting that both cell types could survive using glycolysis for ATP synthesis. However, when hypoxia developed gradually, the majority of OPCs died within 24 h of the onset of the experiment, although astrocytes survived. Interestingly, oxidative stress in OPCs could be prevented either by chelating intracellular iron or by raising the low-background intracellular GSH levels to astrocytic values [36]. Hollensworth and collaborators [37] have noted that astrocytes in primary rat cultures exhibited lower GSH content and SOD activity than did microglia and oligodendrocytes. Using menadione to induce oxidative stress and apoptosis, they observed that the initial mitochondrial DNA (mtDNA) damage was accelerated in the latter. Therefore, they hypothesized that the differential susceptibility of distinct glial cell types to oxidative damage and apoptosis was related to increased oxidative mtDNA damage, allowing the initiation of apoptosis through the enhanced release of cytochrome c and the activation of caspase 9 [37].

The prominent role of astrocytes in GSH metabolism and in defense against ROS in the brain is widely recognized as they supply GSH precursors to neighboring cells in the CNS [38]. Accordingly, excitotoxicity resulting from excitatory amino acids such as glutamate is considered to be a contributing factor in the neuropathogenesis of a number of acute (e.g., stroke, trauma) or chronic CNS disorders [4,36]. Many excitatory substances, such as *N*-methyl-d-aspartate, stimulate massive Ca^2+^ influxes into neurons (and other brain structures) that impose harmful redox imbalances and oxidative stress [39]. The process usually leads to the drastic depletion of cellular thiol (-SH) groups and the activation of nNOS, thereby triggering apoptotic pathways in affected neurons [40]. Therefore, the direct supplementation of the cellular pool of GSH by thiol-enhancing drugs could represent a useful neuroprotective strategy to circumvent –SH (and, thus, general antioxidant) depletion associated with neuropathies. Pretreatments with the GSH-precursor γ-glutamylcysteine (γ-GC) resulted in lower isoprostane formation (biomarker of lipid oxidation) both in neurons and astrocytes, as well as antioxidant gene expression in astrocytes in response to H_2_O_2_-induced oxidative stress [41].

Protein carbonyl formation is an important marker of protein oxidation and can arise from the direct attack of ROS/RNS on some amino acid side chains, from cross-linking reactions with the products of glycation and glycoxidation, or after adduct formation with lipid peroxidation products [42]. Some specifically oxidized proteins have been identified in neurodegenerative brains: (i) creatine kinase (CK), which decreases the ATP supply in synaptic terminals (necessary to drive ion-motive pumps or to synthesize antioxidant proteins); and (ii) glutamine synthetase (GS) and the glutamate transporter Glt-1, which can induce injury in the AD brain via glutamate excitotoxicity, for example. Other markers of neuronal oxidative stress in the mammalian brain include protein oxidation, tyrosine nitration (mainly from ONOO^−^), altered antioxidant enzyme activity, increases in 8-hydroxy-2′-deoxyguanosine (8-OHdG, marker of nucleic acid oxidation), modifications in mtDNA, and altered DNA repair mechanisms [42,43,44,45,46].

One important target for alterations of the cellular redox balance is nuclear transcription factor kappa B (NF-κB), which is implicated in a variety of cell processes, such as inflammation, growth control or apoptosis. It remains uncertain whether the NF-κB response to oxidative stress induces a neuroprotective effect or is deleterious to neuronal survival [31]. Other transcription factors, including c-Myc, c-Fos and c-Jun, are activated by mitochondrial-derived ROS. These transcription factors may, in turn, upregulate protective enzymes like heat shock proteins and antioxidant enzymes [46]. Mitochondrial Mn-dependent SOD, specifically, is thought to be upregulated following TNF-α stimulation of cells via NF-κB. Thus, ROS/RNS cannot be recognized merely as damaging oxidizing species, but also act as small physiological signaling molecules (for example, NO• role in neurotransmission and neuromodulation) [46,47,48].

It is widely recognized that oxidative stress increases with aging, making the brain more susceptible to neurodegenerative disorders. Accumulating data from experimental and human investigations indicate that oxidative stress plays a major role in the pathogenesis of AD [42,49,50,51,52,53,54,55,56,57,58,59,60,61,62,63,64]. The most important hallmarks of AD include neurofibrillary tangles (NFTs), senile plaques, and synaptic loss [42,64]. Amyloid β-peptide [Aβ(1–40) or Aβ(1–42)] is the principal constituent of senile plaques and is generated by the proteolytic cleavage of membrane-associated β-amyloid precursor protein (APP) by the action of β- and γ-secretases. Aβ exists in several aggregated states, among which the oligomeric form of Aβ(1–42) is considered highly toxic as it is inserted into the lipid bilayer [64]. *In vitro* and *in vivo* studies have shown that Aβ induces lipid and protein oxidation, generating extensive formation of ROS/RNS, and inhibiting several neuronal and glial transmembrane transport systems, including ion-motive ATPases, glutamate transporters, glucose transporters, GTP-coupled signaling proteins and polyamine transporters [42]. Lipid peroxidation leads to the production of several aldehydes, including 4-hydroxy-2-trans-nonenal (HNE) and acroleyn (2-propenal), as well as isoprostanes (IsoPs) and the neuronal-specific neuroprostanes (NPs) (from the oxidation of brain PUFAs), which are diffusible and reactive with other biomolecules, increasing their neurotoxicity within the CNS [54,55,56]. Increased levels of such oxidative stress markers are found in the brains of AD subjects with mild cognitive impairment [64].

Neuroinflammation/oxidation is also considered a prominent contributor to the pathogenesis of PD, which includes the progressive loss of dopaminergic neurons in the *substantia nigra* of the midbrain and the depletion of dopamine in the striatum [64,65,66]. Microglia, the resident macrophage-like cells of the CNS that contribute to the innate immune response against microorganism invasion and injury, play a key role in the generation of ROS/RNS [67]. In both PD patients and experimental models of PD in animals, classical features of inflammation are present, including phagocyte activation, increased synthesis and release of proinflammatory cytokines, and complement activation. Given that dopaminergic neurons in the *substantia nigra* are relatively vulnerable to oxidative stress and the region has a larger population of microglia (4.5-fold higher) in comparison to other CNS areas, these events may trigger oxidative neurodegeneration [65]. The oxidative stress hypothesis in PD is supported by the observation of excessive formation of ROS/RNS leading to increased lipid peroxidation, 8-OHdG, GSH depletion, enhanced O_2_^•−^ production, disruption of iron homeostasis, and mitochondrial dysfunction, among other features in PD patients [68].

Amyotrophic lateral sclerosis (ALS) is a devastating disease characterized by the disappearance of motor neurons (through caspase-dependent programmed cell death, resembling apoptosis) causing muscle wasting, paralysis, and death, usually within 2–3 years of onset [69]. It is now accepted that motor neuron death occurs not by a single stimulus, but rather through a combination of mechanisms including glutamate excitotoxicity, mitochondrial dysfunction, endoplasmic reticulum stress and the unfolded protein response, protein aggregation, cytoskeletal dysfunction, glial involvement and defects in RNA processing and trafficking. Oxidative stress is undoubtedly a central mechanism, because ALS patients present elevated markers of oxidative damage in the CNS and cerebrospinal fluid, as well as mutations in the antioxidant enzyme SOD1 in neurons, astrocytes, and microglia in approximately 20% of familial ALS cases [69].

Oxidative stress is also implicated in many other pathological conditions such as X-linked adrenoleukosdystrophy (X-ALD) [70], Huntington’s disease (HD) [71], epileptic seizures [72], CNS vascular diseases [73], HIV-associated dementia [74], major depression disorder [75], schizophrenia [76], and autism spectrum disorders [77], among others.

## 4. Marine Antioxidant/Phytochemical Therapies

The consumption of marine products, including seafood, has been associated with mental health, the prevention of neurodegenerative processes, and the maintenance of cognitive capacities in the elderly. Most of the neurological benefits provided by regular seafood consumption come from an adequate uptake of polyunsaturated fatty acids (PUFAs) and antioxidants. Regarding marine food, omega-3 (*n*-3) and omega-6 (*n*-6) PUFAs, and the antioxidant carotenoid astaxanthin (ASTA) play central roles in oxidative stress in neuronal metabolism. Evidence shows that supplementing the diet with PUFAs—especially in cases with imbalanced ratios of *n*-3/*n*-6 PUFAs—imposes oxidative stress at different *loci*, reflected by reduced levels of antioxidants, e.g., tocopherols and GSH, if supplementation is not accompanied by the administration of adequate antioxidants [78]. Therefore, it is highly necessary to understand the proper mechanisms by which these marine compounds act for or against cognitive and neuromotor health, and how they cope (synergistically?) to improve these functions.

### 4.1. Astaxanthin

Carotenoids are natural pigments provided with regular highly conjugated π-bond systems, and account for the natural yellow, orange, or red colors of many vegetable, fruits, and healthy foods [79]. To date, about 750 naturally occurring carotenoids have been reported [80]. Regarding the behavior-nutritional aspects, carotenoids are directly related to the perception of food quality, both in wild animals and in terms of influencing consumers’ preferences in modern supermarkets [81]. Both the UV/Vis spectrum absorption and the antioxidant properties of carotenoids are fully dependent on the conjugation extension, the electron distribution along their conjugated polyene systems, and the additional presence of hydrophilic groups, such as hydroxyl and oxo groups [82,83].

Animals lack the ability to synthesize carotenoids endogenously and thus need to obtain these important compounds from the diet. Regarding carotenoid chemistry, several authors have postulated that the reactivity and antioxidant properties of carotenoids are fully dependent upon their structure, although their incorporation into membranes, microenvironmental conditions (e.g., pO_2_ in cells), and the chemical nature of oxidizing agents determine their endpoint behavior [84,85].

**Figure 2:**
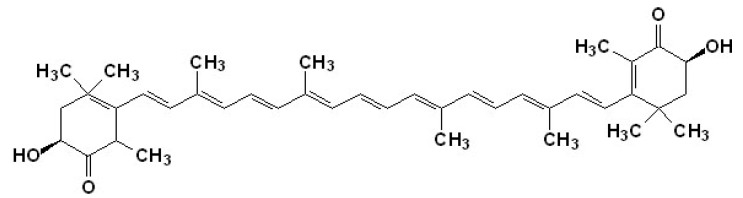
Chemical structure of the marine carotenoid astaxanthin (ASTA).

Astaxanthin (ASTA; Figure 2) is an orange-pinkish carotenoid extensively found in marine organisms, especially salmonid fishes. Humans regularly obtain ASTA from their diet, and the health benefits of ASTA are attributed to its antioxidant and anti-inflammatory activities [86,87,88]. In humans, ASTA has been suggested to perform several beneficial functions including inhibition of PUFA oxidation in membranes, protection against UV-light photooxidation in skin cells, modulation of exacerbated inflammatory responses, control of carcinogenic processes, prophylaxis/regression of stomach ulcers caused by *Helicobacter pylorii* infection, slowing of aging and age-related diseases, and promotion of liver, heart, eye, joint, and prostate health [89,90].

NF-κB promptly responds to oxidative stress and regulates the transcription of genes involved in a wide range of antioxidant, immune, and inflammatory cell functions. While the normal activation of NF-κB is required for cell survival and immunity, inappropriate activation of NF-κB is associated with many ROS/RNS-related human diseases, including cancer, neuroinflammation, and neuronal disorders [91,92,93]. Screening studies of anti-inflammatory natural compounds showed that ASTA significantly inhibits NF-κB activity, an effect that is associated with its major antioxidant activity [94]. These results were also observed in other biological systems and neuroinflammation conditions: (i) ASTA displayed inhibitory effects on several intermediates of the NF-κB cascade (e.g., IKKα/β, IκBα, and NF-κB p65 phosphorylation) in BV-2 microglial cells, suggesting that ASTA modulates interleukin-6 (IL-6) expression via this pathway in activated microglial cells [95]; (ii) ASTA blocked the nuclear translocation of the NF-kB p65 subunit and IKBα degradation, reflecting its inhibitory effect on IKK activity [96]; and (iii) ASTA exhibits anti-inflammatory and anti-cancer effects by inducing apoptosis in induced rat colon carcinogenesis by modulating the expression of NF-κB, cyclooxidase-2, Akt, and extracellular signal-related kinase 2 (ERK-2) [97].

Dietary carotenoids, particularly ASTA, have been repeatedly shown to play a role in regulating the immune response, oxidative damage, and inflammation in humans and animal models [98,99]. Mice supplemented with ASTA showed optimized *ex vivo* splenocyte antibody responses to T-dependent antigens, as well as lymphoblastogenic and cytotoxic activity [100,101]. In humans, dietary ASTA enhanced both cell-mediated and humoral immune responses in young healthy females: improved T-cell and B-cell mitogen-induced lymphocyte proliferation, natural killer (NK) cell cytotoxic activity, interferon-γ, and IL-6 production, and the leukocyte function antigen-1 (an adhesion β2-integrin, LFA-1) expression [102]. Furthermore, since NK cells function in immuno-surveillance against tumors, ASTA may also play a role in cancer etiology [103].

Recently, ASTA was shown to directly affect the oxidative burst triggered in frontline immune cells (neutrophils and lymphocytes) by chemical stimuli [87,88,104]. Evidence shows that ASTA inhibits O_2_^•−^ and H_2_O_2_ production, although it is still unclear if ASTA merely scavenges these ROS or additionally interferes with NADPH oxidase assemblage and/or activation [105]. On the other hand, ASTA effects on NO• generation and oxidative/nitrative modifications on proteins and lipids in immune cells were ambiguous and apparently dependent on ASTA concentration and possible alterations in microenvironmental conditions [106,107]. Because Ca^2+^ homeostasis, NO•, O_2_^•−^, and H_2_O_2_ production (and therefore, that of the powerful oxidizing/nitrating agent ONOO^−^ as well) were directly affected by ASTA treatment, several researchers worldwide hypothesized a possible mitochondrial-centric effect of ASTA in different cell types, a feature that could even affect overall energy and redox cellular metabolism (e.g., via Keap1-Nrf2-ARE). Li *et al.* (2013) [108] recently showed that treatment with ASTA activated the Nrf2-ARE pathway (by inducing Nrf2 nuclear localization) and, consequently, mRNA and protein levels of phase II enzymes (e.g., NQO1, HO-1) were increased via the PI3K/Akt pathway (Figure 1). In fact, many recently published papers reinforce the mitochondrial-centric hypothesis of ASTA action [109,110,111,112]. Moreover, based on the same evidence, apoptotic processes (especially from intrinsic mechanisms) would be remediated by ASTA treatments. Current wisdom on the relationship between ASTA and apoptosis shows conflicting anti- and pro-apoptotic results, depending on ASTA concentrations, cell types, and the concomitant presence of other antioxidants or anti-inflammatory agents [113,114,115,116]. These observations are not surprising due to the well-known dual chemical behavior of carotenoids, as described previously [84,85]. As mentioned earlier, mitochondria redox metabolism and ROS/RNS-mediated mutations on mtDNA have been commonly observed in various neurological disorders, e.g., AD, PD, as well as in natural aging dysfunction [117]. Accordingly, mitochondrial-targeted drugs are now important targets in the field of neurodegenerative diseases [118], and the carotenoid ASTA (isolated or in combination with other compounds) has been strongly suggested to play a role as a putative prophylactic and/or remediation agent against such neuropathies [105,108,119,120,121,122].

### 4.2. Polyunsaturated Fatty Acids (n-3/n-6 PUFAs)

Omega-3 and omega-6 fatty acids, *n*-3 PUFAs and *n*-6 PUFAs, respectively, are termed “essential fatty acids” and are usually obtained from the diet, because they cannot be synthesized by human cells. α-Linolenic acid (ALA) is a *n*-3 PUFA that is endogenously converted into eicosapentaenoic acid (EPA) and subsequently to docosahexanoic acid (DHA). On the other hand, linoleic acid, a n-6 PUFA, can be progressively converted into arachidonic acid (AA), a precursor for several classes of eicosanoids and pro-inflammatory compounds. In the nervous system, cell membranes contain relatively high concentrations of PUFAs, such as docosahexaenoic acid (DHA) [123,124]. The *n*-3 PUFAs are known to play a role in nervous system activity, cognitive development, neuroplasticity of nerve membranes, synaptogenesis, and synaptic transmission [125]. On the other hand, *n*-6 PUFAs (e.g., AA) act as second messengers for pro-apoptotic and inflammatory events. Thus, imbalances in the n-3/n-6 PUFA ratio may result in increased susceptibility to neuronal damage, as observed in neurodegenerative disease [126]. DHA and EPA are mainly found in fish oil (FO) and fatty fish, including salmon, tuna, and trout, whereas ALA is commonly found in vegetable oils such as soya, canola, and linseed oils [127].

Evidence shows that aging and the associated neurodegenerative processes could be influenced by the consumption of EPA and DHA during the lifetime of humans. The dentate gyrus (DG), a sub-region of the hippocampus, is implicated in cognition and mood regulation. The hippocampus represents one of the two areas in the mammalian brain in which adult neurogenesis occurs, with beneficial effects on cognition, mood, and chronic pharmacological treatment. Exposure to DHA and EPA enhances adult hippocampal neurogenesis, which is associated with cognitive and behavioral amelioration, enhanced synaptic plasticity, and the formation of new spines [128]. Consistent with this hypothesis, animals that are fed a diet low in DHA show marked deficits in cognitive function, and those that are subjected to chronic DHA administration show improvement in their learning abilities [129,130,131].

Chronic dietary intake of *n*-3 PUFAs may modulate learning and memory due to the incorporation of these PUFAs into neuronal plasma membranes. However, the effective *n*-3/*n*-6 PUFA ratio is hypothesized to determine membrane fluidity and thereby the function of membrane-bound proteins. With aging, and especially among patients with neurodegenerative diseases, DHA levels in the brain tend to decrease, which suggests that a drop in the *n*-3/*n*-6 PUFAs ratio in brain tissues could contribute to deterioration in memory and other cognitive functions [132,133]. Moreover, the aging process implies morphological and physiological changes in the brain, resulting in higher ROS/RNS production and a decrease in antioxidant capacity. Accordingly, imbalances in the ratio of *n*-3/*n*-6 PUFAs have been implicated in a variety of neurological disorders, ischemic brain injury/stroke, and psychiatric disorders. Neurons lack the enzymes for the *de novo* synthesis of DHA and AA, and hence these substances are obtained from dietary sources or synthesized mainly in the liver from the dietary precursors ALA and LA [134]. However, astrocytes produce DHA, some of which is likely exported to neurons by unknown mechanisms. Several studies have examined the role of astrocyte-generated DHA in neurological disorders [135].

Dietary DHA attenuates Aβ production, AD-like neuropathological changes, and cognitive deficits in a transgenic mouse model of AD [136], and in rats infused with Aβ [137]. The protective role of DHA in AD transgenic mice has been reported in many studies [138,139,140]. However, regarding vascular health, Arendash *et al.* (2007) [141] reported controversial results in AD-like neuropathology or cognition in transgenic mice fed long-term on a diet rich in *n*-3 PUFAs. A review of over 15 prospective cohort studies confirmed the controversy regarding the link between DHA supplementation and AD pathogenesis [142].

In an observational study, Barberger-Gateau *et al.* (2007) [143] evaluated a cohort (*n* = 8085) of patients aged 65 years or older with no dementia (the Three City Cohort Study). After a follow-up period of almost 4 years, they observed that weekly fish consumption presented a correlation with reduced AD risk [hazard ratio (HR) = 0.65, 95% confidence interval (CI) = 0.43–0.994; *p* = 0.047], even after adjusting for body mass index (BMI), and diabetes. Studies using transgenic animal AD models indicate a series of beneficial results in response to *n*-3 PUFA consumption, mainly in terms of synaptic alterations, Aβ accumulation, Tau protein alteration, and cognitive deficits. These models are important for the knowledge of action mechanisms, including the following: positive regulation of neurotrophic factors, inhibition of the inflammatory cascade, reduction in oxidative damage (especially through an increase in glucocorticoid receptor (GR) activity and a reduction in oxidized protein accumulation, lipid peroxidation, and ROS levels), and effects on cellular membrane properties and cellular signaling pathways [144,145]. In agreement, mice with a PD-like phenotype fed on an *n*-3 PUFAs diet for 2–12 months showed significant protection against the decline in dopamine and dopamine transporter mRNA levels when compared to controls, in response to the PD-inducing drug 1-methyl 4-phenyl 1,2,3,6-tetrahydropyridine (MPTP) [146]. In a prospective population-based cohort study of people aged ≥55, the association between PUFA intake and the risk of incident PD was evaluated. Intake of total fat, monounsaturated fatty acids (MUFAs), and PUFAs, was significantly associated with a lower risk for PD [147]. A double-blinded, placebo-controlled study showed that PD patients taking FO, with or without antidepressants, exhibited reduced depressive symptoms, indicating that *n*-3 PUFAs had an antidepressant effect or acted as adjuvant therapy with some other medication for PD [148].

The cellular mechanisms underlying the effects of fatty acids are not clear and should be further investigated in order to understand their effects on neural cells. While most studies have investigated the role of fatty acids on neurons, investigations into their effects on other cell types in the brain (including astrocytes and oligodendrocytes) are sparse. There is also evidence to indicate important roles for specific *n*-3/*n*-6 PUFAss ratios in improving cognitive function and in neuroprotection, but more research and conclusive data are required. A balance between EPA and DHA (3:2, regularly found in natural FO) and putative concomitant participation of antioxidants are apparently key factors for the cognitive and mental health benefits obtained from natural diets, whereas the unaccounted contribution of many other PUFAs—such as pro-inflammatory AA—can, in fact, be harmful [149].

### 4.3. Krill Oil

Evidence shows that ASTA can directly cross the blood-brain barrier (BBB) to reach different mammalian brain regions [111,119,121,150]. Therefore, the pharmacology and pharmacodynamics of absorbed ASTA should also be taken into account when discussing the “neurohormesis” principles in the context of ASTA-rich marine products. Studies from our group demonstrated that the combination of ASTA and FO surpasses the cognitive benefits observed in FO-fed Wistar rats due to an additional antioxidant protection provided at the cathecolaminergic-rich anterior forebrain (associated with anxiety behavior) of the experimental animals [22]. In fact, higher scores of lipid and protein oxidation were observed in the anterior forebrain of Wistar rats when treated only with FO, although positive anxiolytic effects were also observed.

Controversial studies have given rise to the hypothesis that unbalanced *n*-3 PUFA consumption could affect the physicochemical properties of the neuronal membrane (fluidity, permeability, hydrophobicity, *etc.*), thereby impacting the speed of signal transduction and the effectiveness of neurotransmission [151,152,153,154]. Consequently, the neuronal membrane becomes more sensitive to oxidative injury if not properly counterbalanced by antioxidant defenses that sustain the optimal dose-response hormesis ratio [155,156]. The hormesis dose-response principle may thus represent the proper pharmacological strategy to group all these relevant factors into a single drug/phytochemical formulation for pre-clinical studies, clinical trials, and further disease cures. The interplay between *n*-3 PUFAs and antioxidants was investigated early on by Shirai *et al.* (2004) [131], who reported that enhancement of cognitive function was obtained when DHA was supplemented with catechin, a dietary polyphenol. Whether polyphenols and *n*-3 PUFAs act at multiple independent sites to improve cognition is not known and should be further investigated.

The shrimp-like microcrustacean *Euphausia superba Dana* (krill) is one of the most important species among Antarctic marine biota, as it comprises about 50% of the zooplankton biomass [157]. Apart from its pivotal role in micronutrient provision to the upper trophic levels in the Antarctic food web, krill consumption by humans has been increasingly suggested to be a potentially healthy nutrition strategy, especially with regard to immune response enhancement, lowering the risk of cardiovascular diseases, and neuroprotection against progressive cognitive loss [158].

Grantham (1977) [159] reported that krill oil (KO) contains 77.9%–83.1% moisture, 0.4%–3.6% lipids, 11.9%–15.4% protein, and approximately 2% chitin, chitosan, and other glucides [160]. With respect to its lipid content, 30% to 65% of the fatty acids in KO are incorporated into phospholipids, increasing the bioavailability of the lipid components. Additionally, KO also contains high amounts of the powerful antioxidant ASTA [161,162]. Biotechnological studies have shown that depending on the methodology applied for lipid extraction from KO, high amounts of *n*-3 PUFAs and antioxidant activity were obtained, with lower free and esterified cholesterol levels [163]. Of note, the cholesterol content in krill is higher than in fish, but lower than in shrimp [158]. When using these technical procedures, the micronutrient composition of KO would be more suitable for human consumption. Despite its potential as a high-quality lipid, antioxidant, and protein source, the use of krill in human diet has been limited [164]. Few studies have investigated the digestibility and adverse effects of KO uptake by humans. Low-dose supplementation with 2 g KO/day (4 weeks) significantly increased plasma EPA and DHA in overweight and obese men and women and was well tolerated, with no indication of adverse effects on safety parameters [165].

Apart from investigations of bioavailability and absorption, information is also needed regarding the tissue accumulation of lipid components of KO. An extended study performed by Tou and colleagues (2011) [166] showed that salmon oil (SO, also a natural combination of *n*-3 PUFAs and ASTA, but at different ratios than in KO) had the highest deposition of DHA in the whole brain of female rats, although KO-fed animals also had significantly higher brain DHA than FO-fed rats. Regarding *n*-6 PUFAs (high pro-inflammatory activity), brain linoleic acid deposition was highest in FO-fed > KO-fed > SO-fed animals [167]. Interestingly, mice fed KO had higher liver SOD, CAT, and GPX mRNA expression than did lipid-controlled rats, although no information regarding the activation of the Keap1-Nrf2-ARE pathway was provided by the authors [168]. Zucker rats fed with low doses of KO (0.5 g of EPA + DHA per 100 g of diet, equivalent to 0.8% in the rat diet) showed significantly reduced levels of the endocannabinoid 2-arachidonoylglycerol (2-AG) in their brains. Since the dietary treatment did not modify food intake, it is possible that the observed reduction in 2-AG levels did not occur in the hypothalamus (or other brain areas involved in the control of food intake and energy expenditure). Interestingly, KO was able to increase significantly DHA levels in brain phospholipids, with no changes in AA content [169].

Regarding vascular health, high-fat diet-fed C57/Bl6 male mice treated with KO showed a specific decrease in the expression of genes involved in the early steps of isoprenoid/cholesterol synthesis (including HMG-CoA reductase) possibly via the α-, γ-, and δ-peroxisome proliferator-activated receptors (PPARs) [170]. Accordingly, concomitant triglyceride- and cholesterol-lowering effects (free, esterified, HDL-, and non-HDL-cholesterol) were observed for KO feeding in an animal model of persistent low-grade exposure to human TNF-α [171]. Regarding anti-/pro-inflammatory activities, the KO diet tended to decrease MCP-1 in mesenteric adipose tissue, whereas the levels of IL-6 and leptin did not differ substantially between the KO diet and FO or control groups.

Deutsch (2007) [172] reported that most of the important anti-inflammatory effects obtained from relatively short-term KO supplementation (300 mg/day for 7–14 days) in osteoarthritis patients were linked to inhibition of leukotriene formation, deactivation of the lipooxygenase pathway, and possibly to ASTA-mediated inhibition of iNOS activity and IsoP and TNF-α production. Functional impairment scores (WOMAC osteoarthritis scores) confirmed pain relief and lower scales of discomfort following KO treatment. Moreover, cognition (Aversive Light Stimulus Avoidance Test; ALSAT) and depressive-scoring tests (Aversive Light Stimulus and the Forced Swimming Test, UALST and FST, respectively) were assessed in KO-fed rats for 7 weeks (average dose was 0.2 g KO/rat/day). The results showed that KO supplementation significantly improved learning and working memory, displayed significant antidepressant-like effects, and, at the biochemical level, enhanced the mRNA expression of *Bdnf*, a gene implicated in neuronal growth and differentiation [173]. These positive cognition results were further confirmed by Lee and colleagues (2010) [174] who demonstrated that oral administration of 20–50 mg/kg KO to aged rats (with critical deficits in cognitive function, taken as signs of brain neurodegeneration) significantly improved learning and memory retention in the Morris-water maze. Moreover, KO supplementation attenuated the decrease in acetylcholinesterase- and choline acetyltransferase-immunoreactive neurons in the hippocampus, a particularly vulnerable region of the aging brain. Interestingly, lower doses of KO supplementation (20 mg/kg, compared to a 2.5-fold higher dose) resulted in the best-balanced cognition-biochemical benefit in rats, corroborating the “neurohormesis” principle. Most published studies involving KO supplementation focused on the *n*-3 PUFA content (mostly EPA + DHA), with less attention to its relevant ASTA content. Undoubtedly, further studies are necessary to better understand the contribution of this natural combination of an adequate mixture of anti-inflammatory PUFAs and a powerful antioxidant carotenoid.

## 5. Conclusions

Based on oxidative stress and neuroinflammatory concepts, a critical key to successfully intervene via nutrition against the initiation/progression of neurodegenerative diseases and cognitive impairment is getting the right dose that will afford an adequate redox balance in the highly oxidation-sensitive regions of the human brain. On the other hand, genetic profile, age, gender, diet, sedentary habits, lack of exercise, tabagism, and health status could apparently provide varied redox statuses in plasma, brain tissues and other biological matrices. The “neurohormesis” principle, supported by accurate physiological information from (preferably non-invasive) redox biomarkers may thus dictate pharmacological strategies to create specific formulations that could group all these relevant factors for the efficient treatment of neuronal or CNS-related diseases. Further studies are necessary to better identify the hallmarks of brain redox impairment at different stages of progressive neurodegenerative disease.
